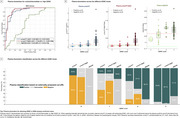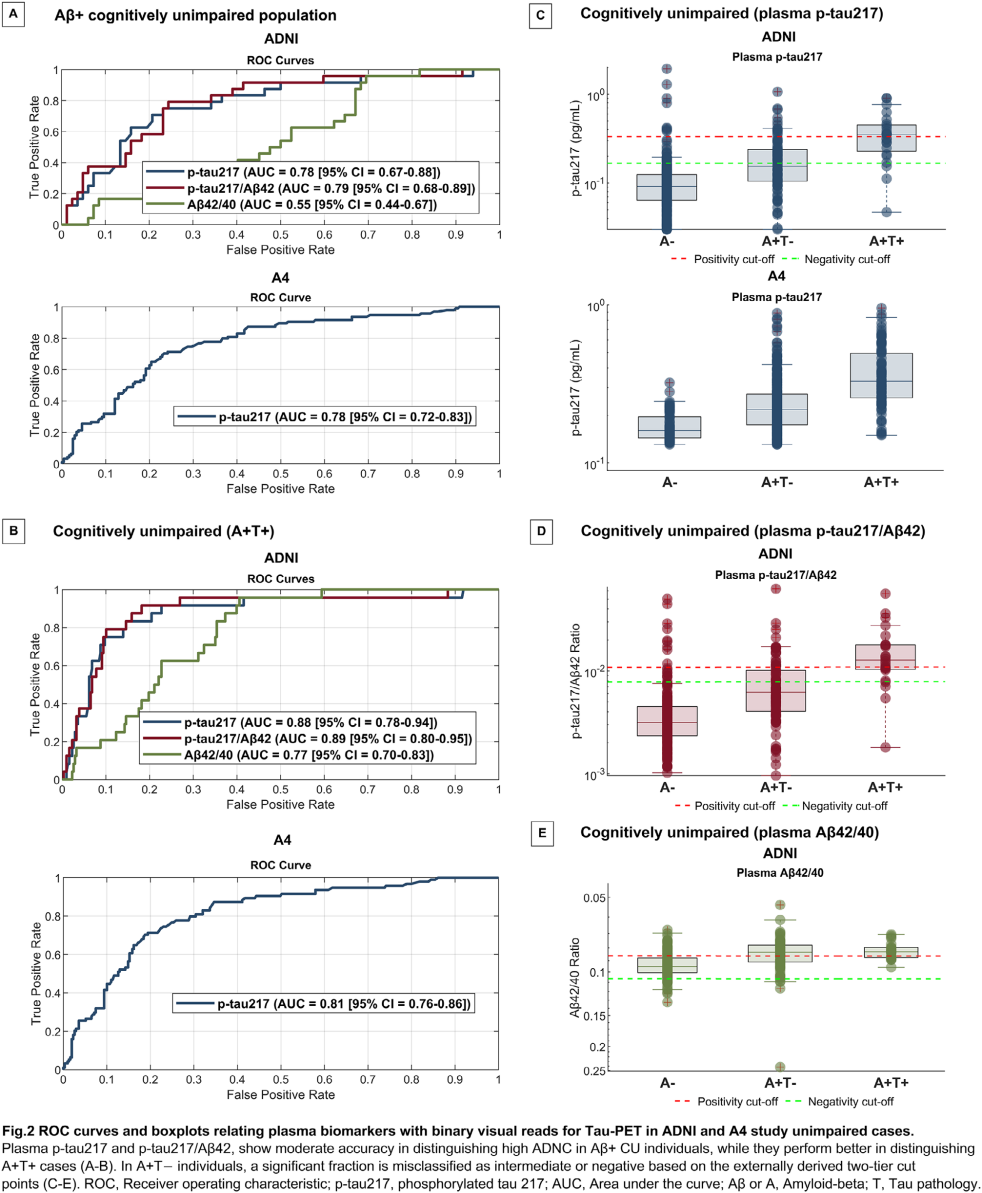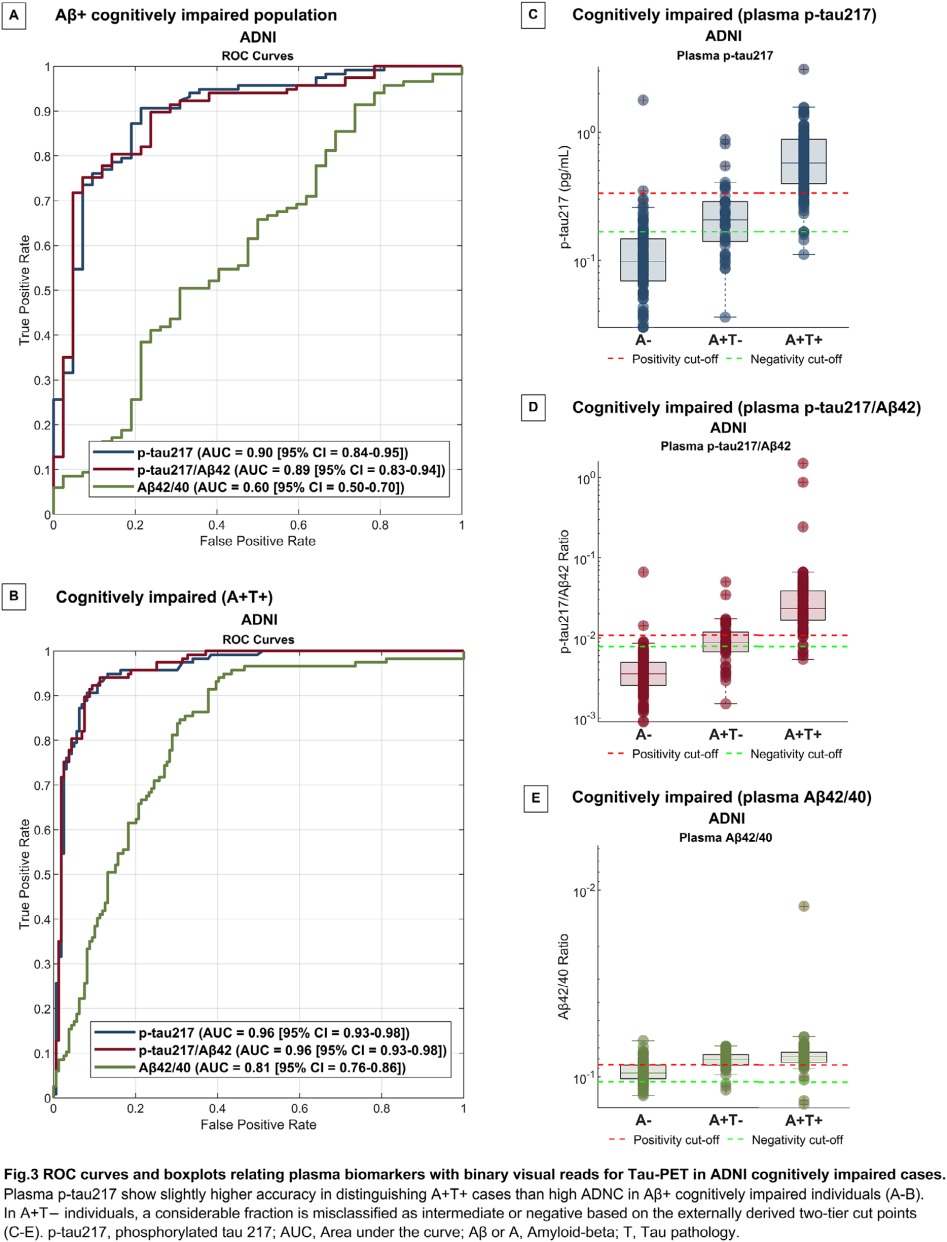# Differential performance of plasma *p*‐tau217 in detecting intermediate vs high Alzheimer's disease neuropathologic change

**DOI:** 10.1002/alz70856_104257

**Published:** 2025-12-26

**Authors:** Stamatia Karagianni, Alexis Moscoso Rial, Martijn van Essen, Ismini Mainta, Valle Camacho, Omar Rodríguez‐Fonseca, Andrés Perissinotti, Michel J. Grothe, Valentina Garibotto, Michael Schöll

**Affiliations:** ^1^ Department of Psychiatry and Neurochemistry, Institute of Neuroscience and Physiology, The Sahlgrenska Academy, University of Gothenburg, Gothenburg, Sweden; ^2^ Wallenberg Centre for Molecular and Translational Medicine, University of Gothenburg, Gothenburg, Sweden; ^3^ Instituto de Investigación Sanitaria de Santiago de Compostela, Santiago de Compostela, Travesía da Choupana s/n, Spain; ^4^ Department of Neuropsychiatry, Sahlgrenska University Hospital, Gothenburg, Sweden; ^5^ Division of Nuclear Medicine, Geneva University Hospitals, Geneva, Switzerland; ^6^ Nuclear Medicine Department, Hospital de la Santa Creu i Sant Pau, Barcelona, Barcelona, Spain; ^7^ Nuclear Medicine Department, Lucus Augusti University Hospital (HULA), 27003 Lugo, Spain., Lugo, Galicia, Spain; ^8^ Centro de Investigación Biomédica en Red de Fragilidad y Envejecimiento Saludable (CIBERFES), Instituto de Salud Carlos III, Madrid, Spain; ^9^ Nuclear Medicine Department, Hospital Clínic i Provincial de Barcelona ‐ Universitat de Barcelona, Barcelona, Spain; ^10^ CIEN Foundation, Reina Sofia Alzheimer Center, ISCIII, Madrid, Madrid, Spain; ^11^ Centro de Investigación Biomédica en Red sobre Enfermedades Neurodegenerativas, Instituto de Salud Carlos III, Madrid, Spain; ^12^ Faculty of Medicine, University of Geneva and Center for Biomedical Imaging (CIBM), Geneva, Switzerland; ^13^ Dementia Research Centre, Queen Square Institute of Neurology, University College London, London, United Kingdom

## Abstract

**Background:**

In vivo detection of intermediate and high Alzheimer's disease neuropathologic changes (ADNC) is key for disease staging and clinical trial enrolment. While a visually positive tau‐PET scan with the FDA/EMA‐approved tracer [^18^F]flortaucipir can be considered a reliable proxy of high ADNC (Fleisher et al., 2024), plasma biomarkers such as tau phosphorylated at threonine 217 (*p*‐tau217) are emerging as minimally‐invasive alternatives. This study evaluates the performance of commercially‐available plasma *p*‐tau217 in identifying intermediate and high ADNC defined by neuropathologic assessments at autopsy as well as by visual reads of FDA/EMA‐approved in vivo tau‐PET scans.

**Method:**

Postmortem neuropathologic evaluations and antemortem plasma *p*‐tau217 and Aβ42/Aβ40 measurements (Lumipulse G1200 assay) from 110 ADNI participants were included. In addition, plasma biomarkers were assessed in 625 additional ADNI participants and for 403 A4 participants plasma *p*‐tau217 (MesoScale ECL assay) was included. All participants underwent [^18^F]flortaucipir PET. Three trained readers rated the [^18^F]flortaucipir scans as visually positive/negative using the FDA/EMA‐approved interpretation method. Receiver operating characteristic (ROC) curve analysis assessed the biomarkers’ discriminative accuracy across ADNC levels.

**Result:**

Plasma *p*‐tau217 biomarkers demonstrated high accuracy (AUC>0.93) for discriminating between “no/low/intermediate” and “high” ADNC at autopsy (Figure 1A). When applying externally derived two‐tier cut‐points for plasma *p*‐tau217 (negative/intermediate/positive), it identified “no” or “high” ADNC accurately (80‐93%) (Figure 1B‐C). However, for detecting low/intermediate ADNC, high variability was observed, with only 40‐65% of the low ADNC cases classified as negative and 35‐57% of the intermediate ADNC cases classified as positive (Figure 1C). Findings in the in vivo cohort examined with tau‐PET supported these results. Plasma biomarkers reliably identified high ADNC as reflected by the PET‐based A+T+ profile, but the majority of the A+T‐ individuals were classified as intermediate or negative (Figure 2C‐E and Figure 3C‐E). Plasma biomarkers yielded higher accuracy in cognitively impaired (Figure 3A‐B) than in unimpaired individuals (Figure 2A‐B).

**Conclusion:**

Plasma *p*‐tau217 markers effectively classify ADNC extremes but are less reliable for accurately assessing low/intermediate ADNC levels, reflecting their limited utility for biological disease staging. This weaker performance in preclinical cases, including failure to reliably identify A+T‐ cases as positive, challenges their potential role in early AD detection. Complementary imaging may be needed to improve reliability in early stages.